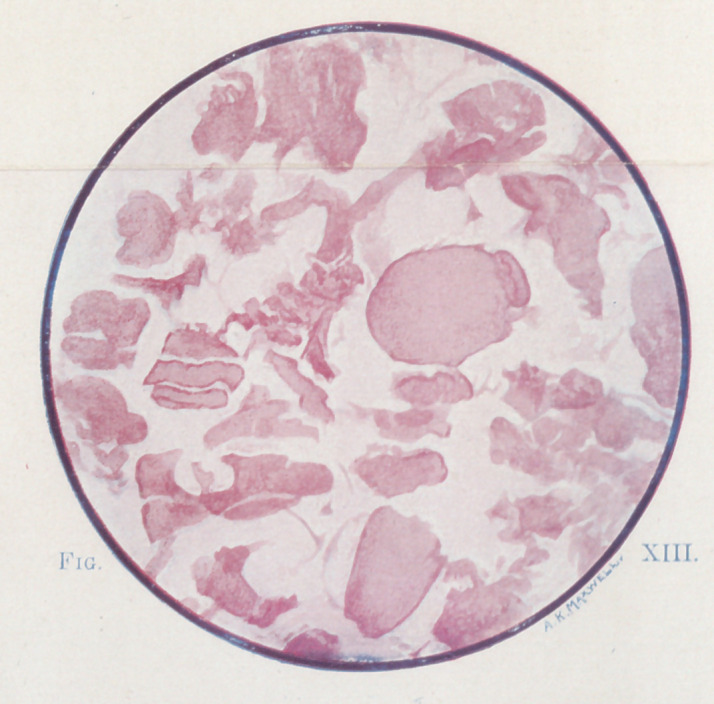# The Color Changes Seen in Skin and Muscle in Gas Gangrene

**Published:** 1917-12

**Authors:** 


***The Color Changes Seen in Skin and Muscle in Gas Gangrene.*** By Col. Cuthbert Wallace, C. M. G., A. M. S. Abs. from the *British Medical Journal*, June 2, 1917.

W. declares himself confirmed by later experience in his support of Taylor's contention that gas gangrene is primarily and mainly a disease of muscle. (See p. 109.) He now adds to his previous obser-

REPRINTED BY COURTESY OF THE BRITISH MEDICAL JOURNAL

FIG. I

GAS GANGRENE.

[ REPRINTED BY COURTESY OF
THE BRITISH MEDICAL JOURNAL

FIG. II.

FIG. III.

FIG. IV.

FIG. V

FIG. VI.

FIG. VII.

FIG. VIII.

FIG. IX

FIG. X.

FIG. XI.

FIG. XII.

FIG. XIII.

vations a description of the naked-eye alterations in the appearances of the skin and muscle in the sequence in which they occur. The colored drawings which accompany the article, and which are here reprinted by courtesy of the *British Medical Journal*, were made by Sergeant A. K. Maxwell, R. A. M. C.

*Color Changes in the Skin.* The icteric tint that affects the whole body in some cases may appear on the skin of a gangrenous arm. In the neighborhood of the lesions the skin, in the early stages, is usually perfectly normal; even under a normal skin the infection may be so far advanced as to necessitate amputation. The first change in color is due merely to the swelling. The skin appears tense and somewhat whiter than normal, owing to interference with the circulation. There may be resonance and crepitation at this stage. A dirty cream tint gradually appears and may be taken as a sure sign that gangrene is established. Up to this point probably only a single muscle or limited group of muscles is involved, and local excision may suffice by way of treatment.

" Subsequent skin changes are quicker and more dramatic. Areas of purple staining appear, which enlarge and coalesce. The margins of these are fairly distinct but irregular, and the intervening skin is grayish-white in color. Soon there appear blebs filled with fluid which is stained by altered blood; removal of the cuticle from these exposes a shiny purple-red area of dermis. When this condition of the skin is reached it may safely be inferred that the gangrenous process is so far advanced as to necessitate amputation. "

As to the yellow-green tint that appears in a later stage of the infection, W. considers it the same as that which appears in any limb after death, and therefore a *post mortem* condition due either to bacterial action or the stoppage of the blood supply. Hence the yellow-green tint sometimes appears early in the neighborhood of the infected wound, although the purple tint is still to be seen at the time of operation over parts of a limb infected later because of the stoppage of blood supply.

In Figure 1 the drawing represents an arm amputated because of gaseous gangrene arising in a wound which had caused great destruction of muscle, but which had not interfered with the main channels of circulation. The spread of the infection was not, therefore, due to the stoppage of the blood supply, but merely to the direct infection of the various muscles, which were themselves destroyed as well as the skin and the subcutaneous tissues. W. calls attention to the purple mottling of the skin, and to the contrast between the infected flexor muscle seen through the wound and the normal-colored muscle by the side of the cut humerus.

The " bronzing " often mentioned in descriptions of the disease, W. finds more difficult to understand. He has been unable to procure a colored drawing of it, although he cites one already published (*British Journal of Surgery*, Vol. VI, No. 13, p. 57). In his experience " bronzing " has appeared more often on the body than on the limbs. He has noted it especially in connection with wounds of the extraperitoneal part of the colon. It may or may not accompany subcutaneous emphysema. It appears over tissues sometimes normal, sometimes slightly edematous, sometimes distinctly edematous, and sometimes yellow-green. Sometimes it appears over violently infected regions that must be excised : at other times it will disappear without such treatment. W. is at a loss to explain its cause.

*Color Changes in the Muscles*. Figure 2 shows the appearance ot a muscle that has been killed by gaseous gangrene. It will be noted that the injury was chiefly upon the vastus externus, and that the damaged part and the subcutaneous tissue was of a dirty yellowish green hue. This muscle, however, had not become infected as a whole, because, as W. thinks, the wound was of an open nature. The bullet had passed undert he rectus tendon and had lodged against the fibers of the vastus internus. This last muscle became gas-gangrenous throughout. The change in color to a brick red is easily apparent in the drawing. The whole muscle was dead and non-contractile. W. calls this the stage of " red death ". Bubbles of gas were visible between the fasciculi. Although it does not clearly appear in the drawing, the outer edge of the lower part of the sartorius, where it lies in juxtaposition to the vastus internus, was also infected. Other muscles of the thigh were not affected. The patient died in this case fifty-six hours after the wound was received. The intermuscular tissue showed but little alteration. W. notes that this is often the case, althrough at times there are distinct alterations. Gas may be found abundantly along the great vessels, and commonly in the subcutaneous tissues, although cultures from such gaseous tissues may prove sterile. At other times the connective tissue is edematous but not discolored, and it sometimes is gelatinous. Later the edematous tissue becomes yellow or yellowish-green, but remains transparent.

Figure 3 is a notably good illustration of a case in which only one muscle, the supinator longus, is involved. This muscle shows a diffluent stage of infection, it is yellowish-green and quite devoid of form or tone. It is soft and can be moulded or dented with the finger. W. calls attention especially to the striking changes that have taken place in the subcutaneous tissue, which is greenishyellow and edematous, although before the skin was deflected, the external appearance was not marked. If the general condition of the patient had been good, excision might have sufficed.

The small fragments of muscle (Figures 4, 5, 6, and 7) show the color changes with considerable detail. 4, 5, and 6 are bits of the gluteus maximus muscle. 7 is of a tibialis anticus muscle. 4 represents perfectly healthy muscle. 5 is of muscle which is dead, non-contractile, crepitant, and brick-red in color. The pockets occupied by gas bubbles can be seen. The muscle in this state is easily friable, and the gas by stroking can be pushed from place to place through the fibers. This is the stage the author calls " red death ". Between figures 4 and 5 a condition that is impossible to portray intervenes. W. describes it as follows : " At the limit of the advancing gangrene, the muscle is simply lighter in color than normal, but not definitely red. It is firmer in consistency than the normal, and no crepitation can be made out. "

Figure 6 represents a further stage of the infection. The color has changed from brick-red to olive-green. The tissue is more friable and it has the consistency of putty.

Figure 7 represents an end stage not often seen in operation. The color is greenish black and the surface glistening. It is so soft that it flattens out when placed on a hard surface.

The condition of the supinator longus in figure 3 is intermediary between that of figure 6 and that of figure 7.

## Figures and Tables

**Fig. I f1:**
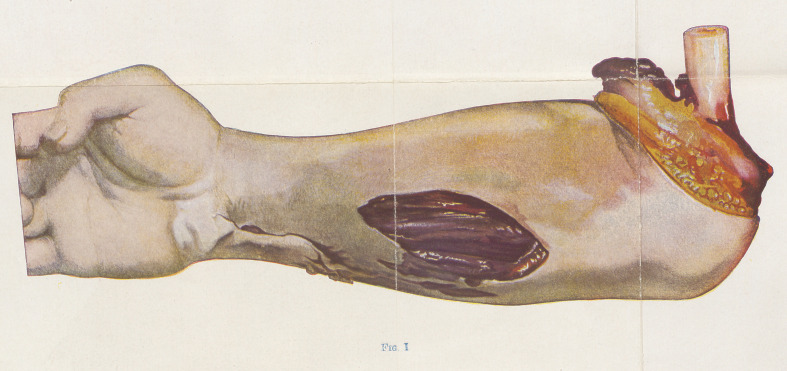


**Fig. II. f2:**
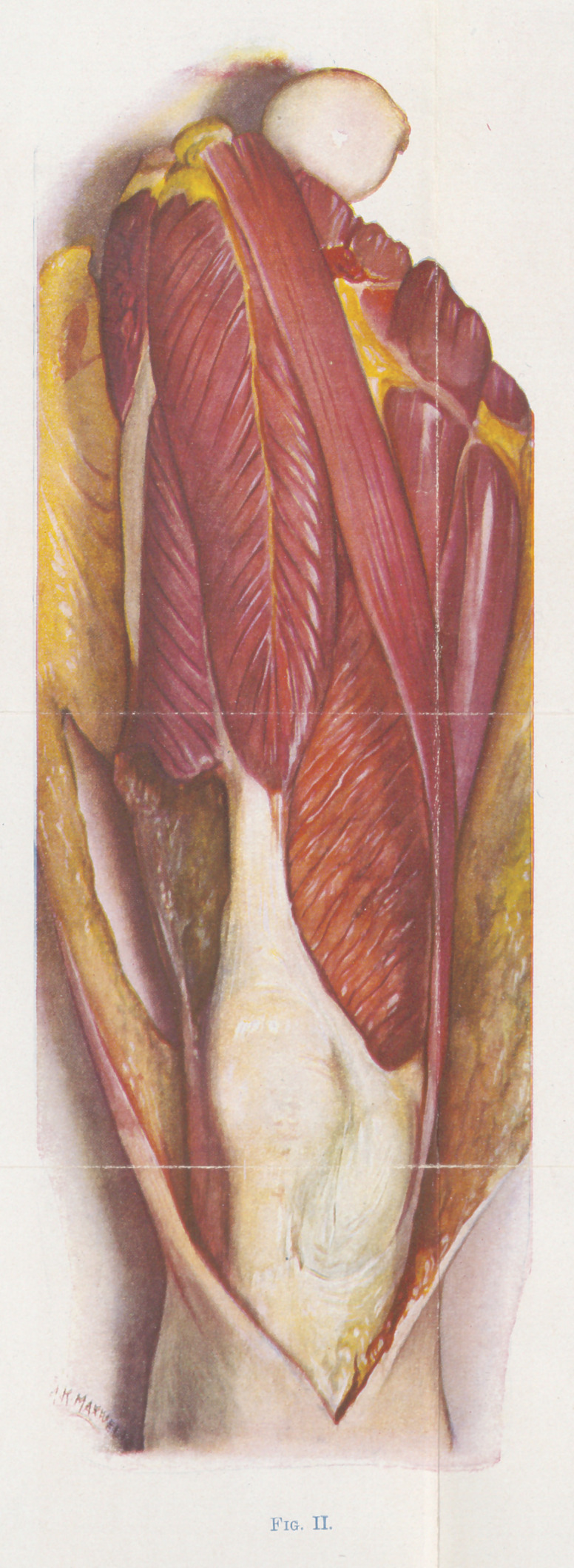


**Fig. III. f3:**
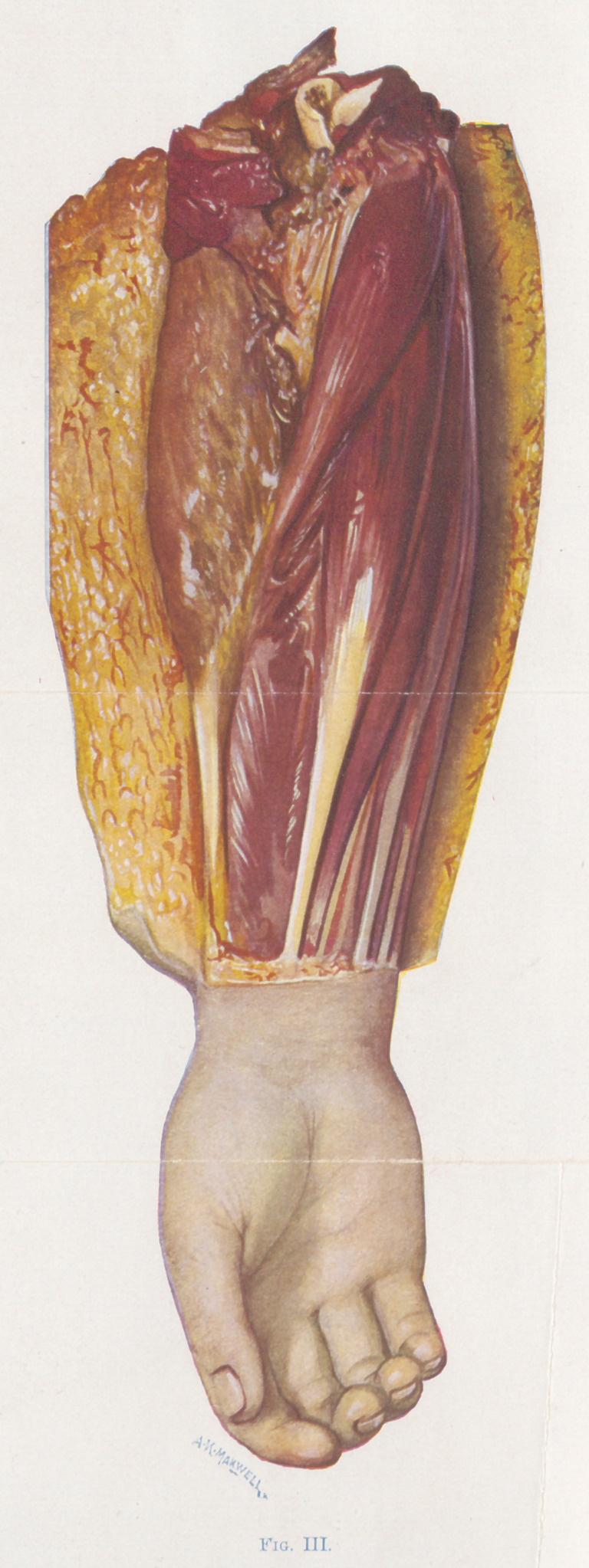


**Fig. IV. f4:**
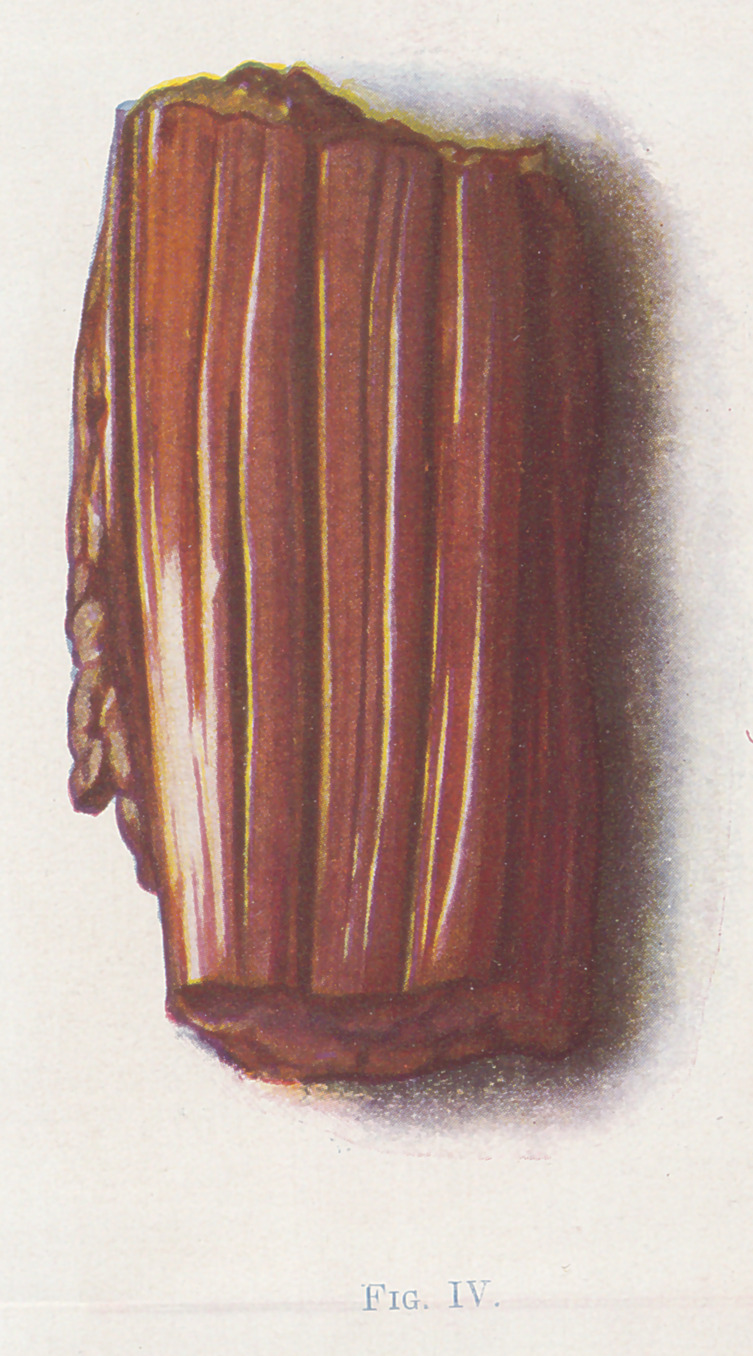


**Fig. V f5:**
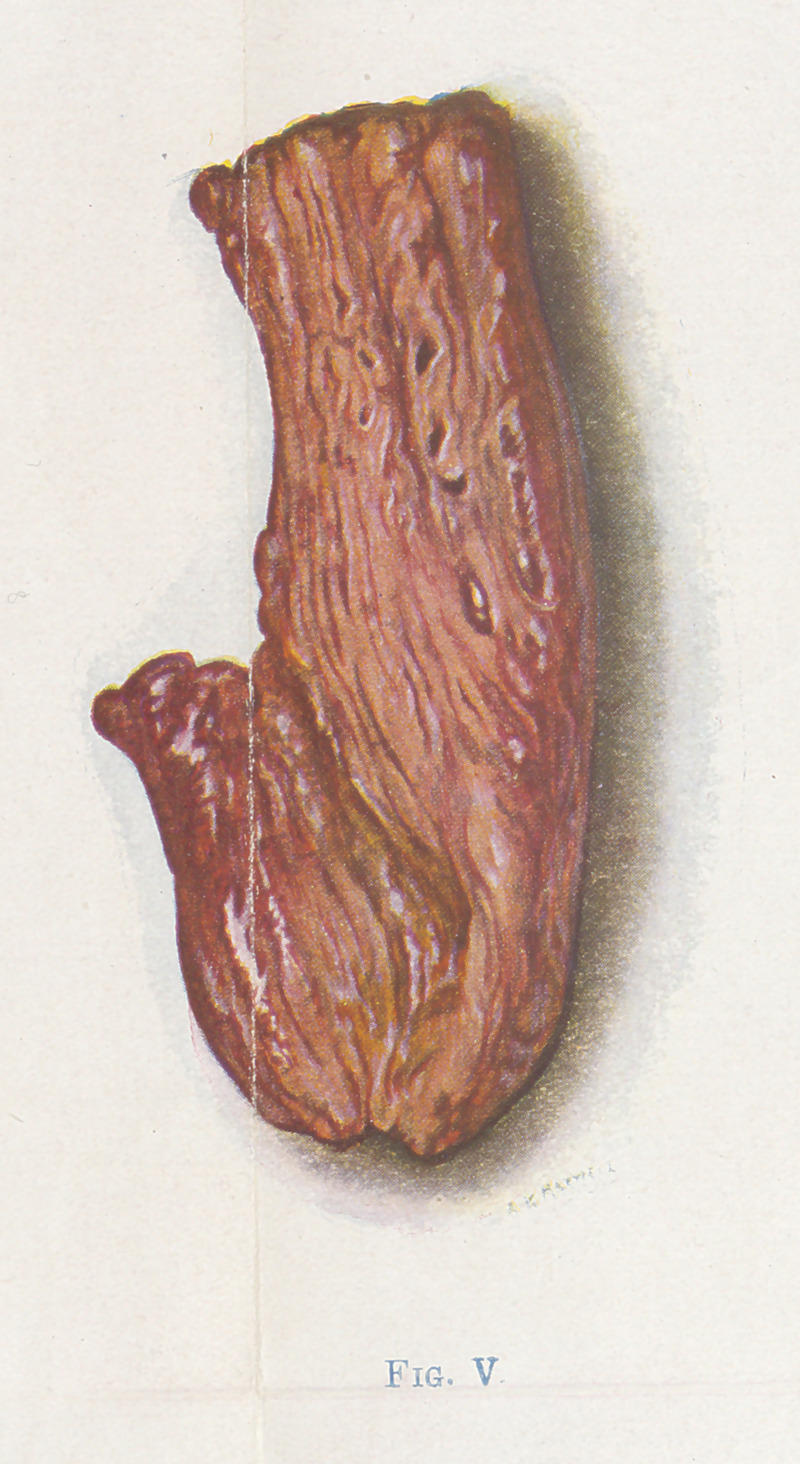


**Fig. VI. f6:**
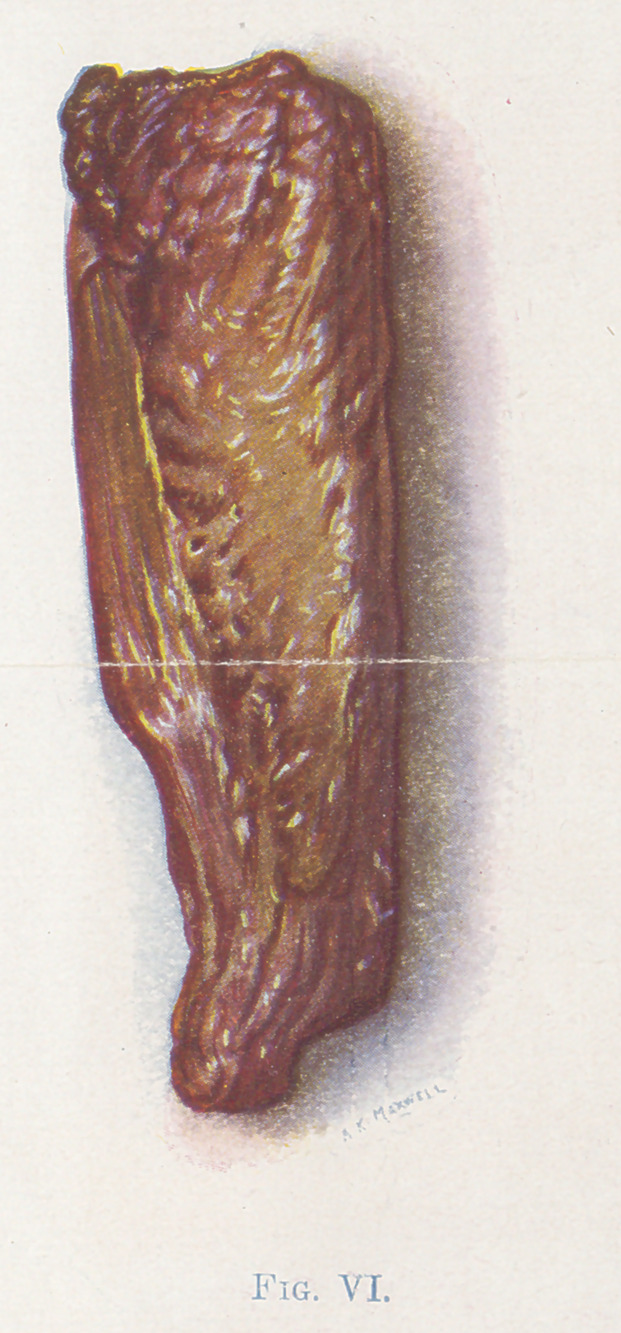


**Fig. VII. f7:**
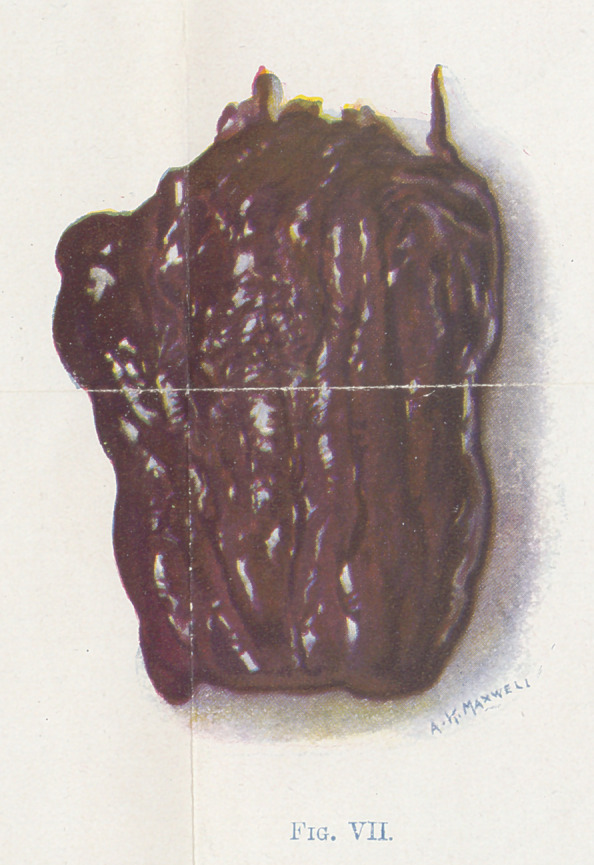


**Fig. VIII. f8:**
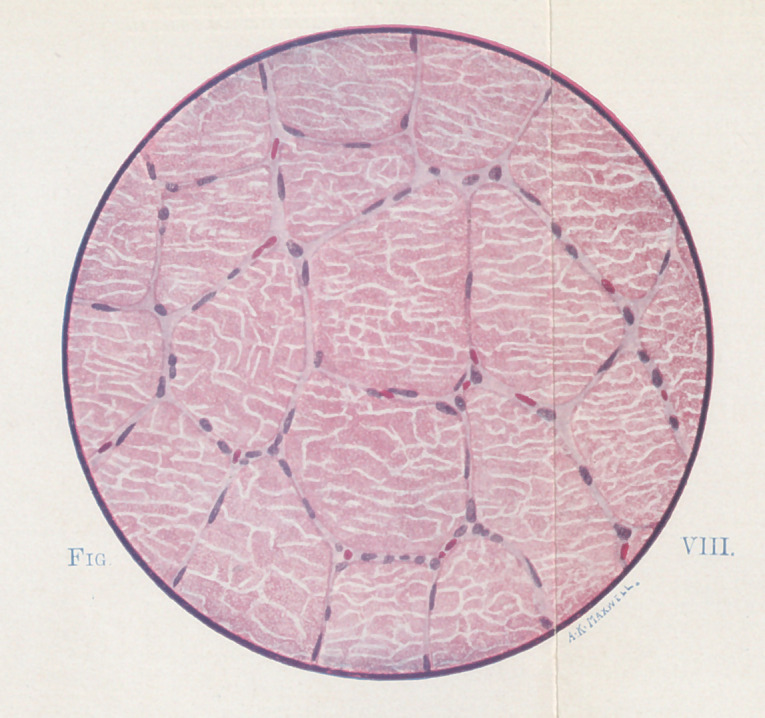


**Fig. IX f9:**
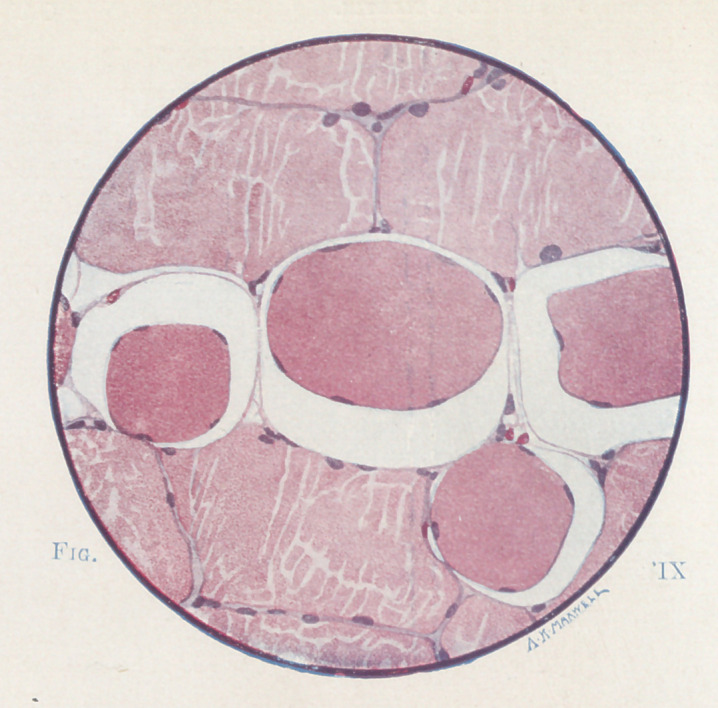


**Fig. X. f10:**
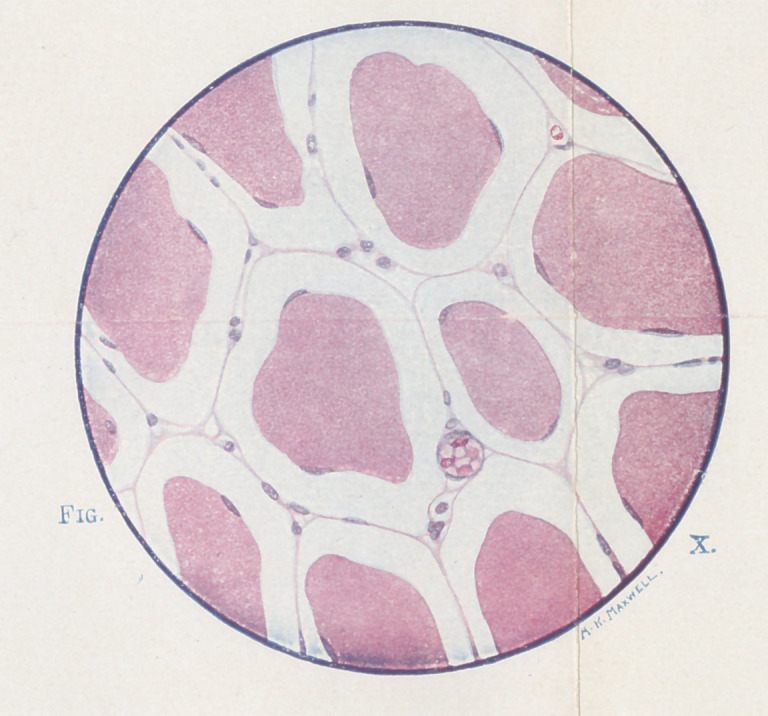


**Fig. XI. f11:**
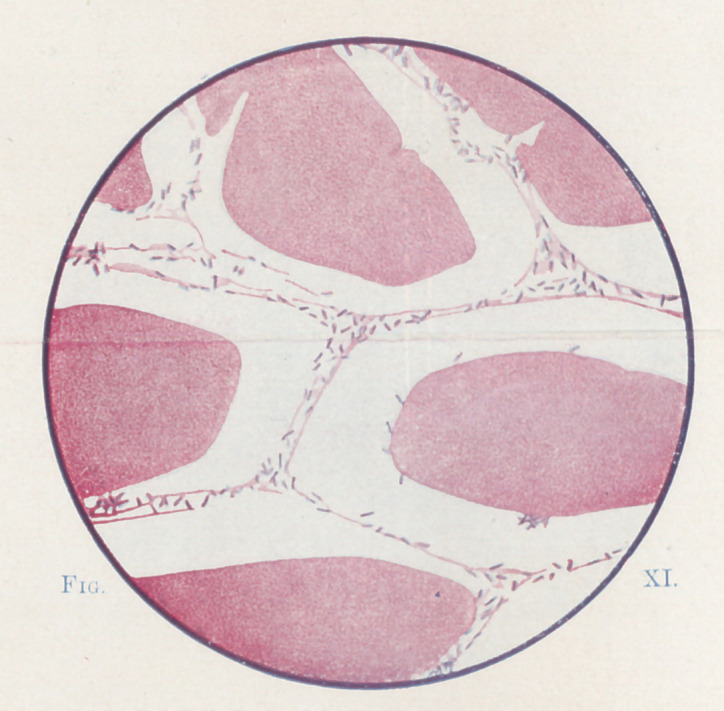


**Fig. XII. f12:**
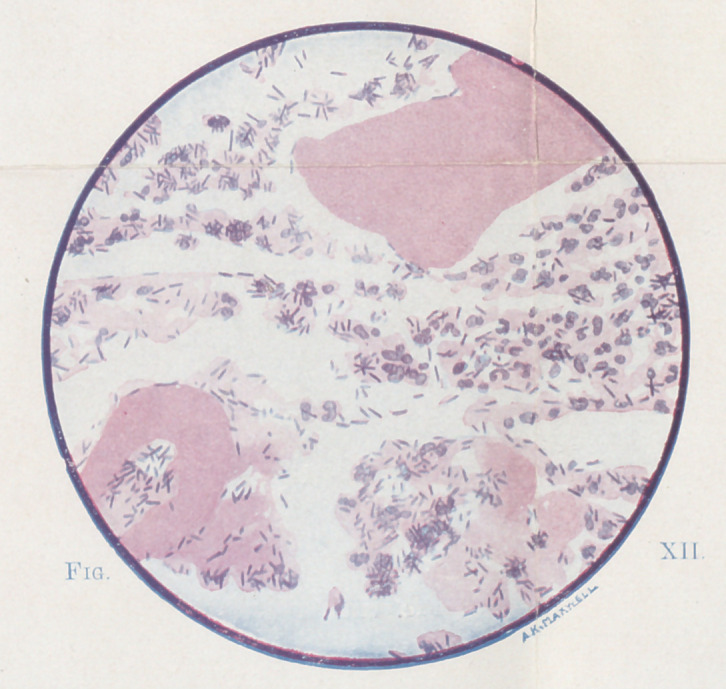


**Fig. XIII. f13:**